# Development and content validity of a rating scale for the pain and disability drivers management model

**DOI:** 10.1186/s40945-022-00137-2

**Published:** 2022-05-16

**Authors:** Florian Naye, Simon Décary, Yannick Tousignant-Laflamme

**Affiliations:** 1 School of Rehabilitation, Faculty of Medicine and Health Sciences, Université de Sherbrooke, 3001 12e Avenue Nord, Sherbrooke, Qc J1H 5N4 Canada; 2 Clinical Research of the Centre Hospitalier Universitaire de Sherbrooke (CRCHUS), Sherbrooke, Qc J1H5N4 Canada

**Keywords:** Low back pain, Assessment, Rehabilitation, Biopsychosocial, Patient-centered care

## Abstract

**Background:**

Establishing the biopsychosocial profile of patients with low back pain (LBP) is essential to personalized care. The Pain and Disability Drivers Management model (PDDM) has been suggested as a useful framework to help clinicians establish this biopsychosocial profile. Yet, there is no tool to facilitate its integration into clinical practice. Thus, the aim of this study is to develop a rating scale and validate its content, to rapidly establish the patient’s biopsychosocial profile, based on the five domains of the PDDM.

**Methods:**

The tool was developed in accordance with the principles of the COSMIN methodology. We conducted three steps: 1) item generation from a comprehensive review, 2) refinement of the scale with clinicians’ feedback, and 3) statistical analyses to assess content validity.

To validate the item assessing with Likert scales, we performed Item level-Content Validity Index (I-CVI) analyses on three criteria (clarity, presentation and clinical applicability) with an a priori threshold of > 0.78. We conducted Average-Content Validity Index (Ave-CVI) analyses to validate the overall scale with a threshold of > 0.9.

**Results:**

In accordance with the PDDM, we developed a 5-item rating scale (1 per domain) with 4 score options. We selected clinical instruments to screen for the presence or absence of problematic issues within each category of the 5 domains. Forty-two participants provided feedback to refine the scale’s clarity, presentation, and clinical applicability. The statistical analysis of the latest version presented I-CVI above the threshold for each item (I-CVI ranged between 0.94 and 1). Analysis of the overall scale supported its validation (Ave-CVI = 0.96 [0.93;0.98]).

**Conclusion:**

From the 51 biopsychosocial elements contained within the 5 domains of the PDDM, we developed a rating scale that allows to rapidly screen for problematic issues within each category of the PDDM’s 5 domains. Involving clinicians in the process allowed us to validate the content of the first scale to establish the patient’s biopsychosocial profile for people with low back pain. Future steps will be necessary to continue the psychometric properties analysis of this rating scale.

**Supplementary Information:**

The online version contains supplementary material available at 10.1186/s40945-022-00137-2.

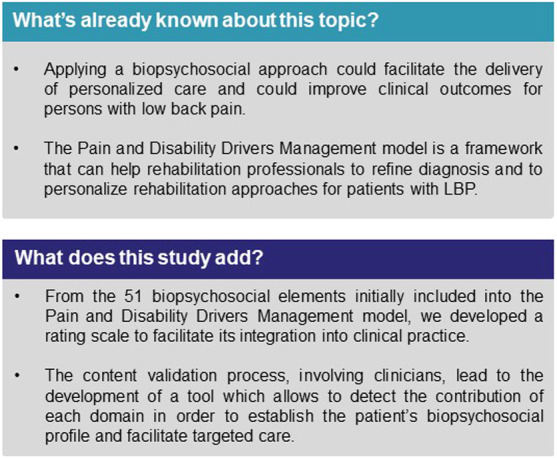



## Introduction

People presenting with low back pain (LBP) display heterogeneous physical, psychological, and social characteristics [1]. Recognizing such heterogenous profiles has led to several approaches attempting to divide this population into homogeneous subgroups [2]. To facilitate the delivery of more tailored physiotherapy interventions, classification systems were proposed as a means to stratify care according to the patient’s profile [3]. However, utmost classification systems poorly incorporate a biopsychosocial perspective, as most are driven by mechanical factors [4]. Therefore, there is a need to develop and propose biopsychosocial stratification approaches to appreciate the complexity of each clinical presentation [1, 5].

As a potential solution to the problem, our team developed the Pain and Disability Drivers Management (PDDM) model — a biopsychosocial diagnostic framework that encompasses the multidimensional elements included within the International Classification of Functioning, Disability and Health framework [6]. This model aims to identify the domains influencing pain and disability to establish the patient’s biopsychosocial profile (or phenotype) [6]. This structure has the potential to help clinicians identify, organize, and facilitate characterizing complex cases of LBP and ultimately, to provide targeted care [7].

The PDDM model includes five biopsychosocial domains known to drive pain and disability in patients with LBP: a) Nociceptive pain drivers, b) Nervous system dysfunction drivers, c) Comorbidity factors, d) Cognitive-emotional drivers, and e) Contextual drivers [6]. To capture the complexity of LBP, each domain is divided into two categories. The first category (category A) relates to relatively common and modifiable drivers of pain and disability, whereas the second category (category B) contains more complex and/or less modifiable elements [6]. These non-mutually exclusive categories allow to weigh the relative contribution of each domain in the patient’s profile, where the elements contained in the model and their allocation within categories were validated by a panel of clinicians and/or researchers with expertise in pain management [8].

More recently, we determined the applicability of the PDDM model and explored clinicians’ perceived acceptability of its use in clinical settings, where 24 clinicians were trained to apply the PDDM model to guide their management of 61 patients [9]. The model contributed positively to the biopsychosocial assessment and better understanding of the psychosocial factors [9], which facilitated the development of a personalized management plan, including a referral process to another professional when deemed necessary [9].

As the PDDM model showed, it can be utilized to overcome certain barriers associated with the integration of a biopsychosocial perspective in clinical practice [10–13] and induced positive changes on various clinical outcomes [9].

However, further clinical integration of the PDDM model requires a comprehensive assessment. Thus, the aim of this study is to develop and validate a rating scale that allows to determine the contribution of each domain of the PDDM model. The specific objectives are to: 1) Generate items to develop an initial rating scale, 2) refine the initial version of the scale with clinicians’ feedback, and 3) assess the content validity of the latest version of the rating scale with statistical analyses.

## Methods

The Consensus-based Standards for the selection of health Measurement INstruments (COSMIN) proposes a risk of bias checklist for patient-reported outcome measurements [14]. This checklist includes boxes for every step of development, validity assessment, reliability assessment, and responsiveness assessment. We relied on the proposed standards for the development and content validity, which includes three steps.

### Step 1: item generation to develop an initial rating scale

#### Definition of the conceptual framework and objective of the rating scale

The PDDM model, described in detail elsewhere [6], served as the theoretical framework upon which the tool was constructed. The feasibility trial provided evidence for the relevance of establishing the patient’s profile according to the presence or absence of the categories within each domain [9]. Thus, we developed a 5-item rating scale (1 item for each domain) to detect the presence or absence of these categories. This allows to determine the contribution of each domain.

#### Operationalization of the rating scale

Screening for the contribution of the categories of each domain involves determining the presence of clinical characteristics (elements) within each category (A and/or B). However, the PDDM model is comprised of 51 different elements [8]; determining the presence/absence of every single element is not feasible in clinical settings [15]. We solved this problem by developing a rating scale able to rapidly detect the contribution of each category. We then developed a scoring method which remained coherent with the objective of the rating scale (i.e., determine the contribution of each domain) and the structure of the domain (i.e., separation into categories A and B).

#### Item generation

To generate items, we used the results of our previous Delphi study [8], which identified clinically relevant elements for each category. From the list of 51 elements distributed into the domains/categories, we performed a comprehensive literature review to determine the most appropriate screening tool(s)/clinical procedures to screen for the presence of elements within each domain/category. More details on the comprehensive literature review are available in the Supplementary Material section. Following this review, we selected the tools/clinical procedures based on guiding principles from some of the barriers found in an implementation of outcome measures in outpatient rehabilitation settings [15]. The guiding principles included: 1) Time to complete, 2) the need for equipment, 3) the clinical utility (e.g., a self-questionnaire is more relevant than a test that requires a 30-min procedure), 4) the usual clinical procedures (e.g., the procedures of the neurological examination are known and well disseminated), 5) clinicians’ knowledge about the measured key characteristic, and 6) the available psychometric data. This process enabled us to generate an initial version of the scale.

### Step 2: content analysis to refine the rating scale

The objective of this step was to obtain participants’ written feedback on the initial version of the scale and refine the content of the rating scale.

#### Recruitment of participants

We recruited physiotherapists, with no prior exposure to the PDDM, who participated in a one-day workshop about the integration of the PDDM in clinical practice. Those clinicians previously registered for one of five workshops offered by the College of Physiotherapy of Quebec *(Ordre Professionnel de la Physiothérapie du Québec*). Details pertaining to the workshop can be found in Appendix 1. This recruitment strategy allowed us to maximize participant variability (different settings, background, practice profile). Inclusion criteria for participating in this study were: (1) being a licensed physiotherapist, (2) participating in the one-day workshop pertaining to the PDDM model, and (3) providing consent for use of data gathered within the context of this project. The Ethics Review Board of the Research Center at the Centre Hospitalier Universitaire de Sherbrooke (project #2021–3440) approved this study.

#### Procedures and analysis

During the last segment of the workshop, the participants were given the initial version of the rating scale and were asked to use it to analyze two clinical vignettes. They then provided written feedback on the difficulties encountered and on the rating scale’s clarity and presentation. The two clinical vignettes were developed according to the framework of Skilling and Sylianides [16] - the vignettes are available elsewhere [17]. For each item of the scale, we collected the participants’ feedback using a comments and suggestions section. The feedback provided was analyzed and used to refine the scale.

The analysis of the participants’ feedback and the modification process (update) involved: a) categorizing comments and suggestions based on difficulties encountered, clarity or presentation, b) interpreting comments and suggestions to determine potential modifications, and c) applying the most parsimonious modifications to meet participants’ needs without content and/or visual overload. Then, the new (updated) version was evaluated by the participants of the following workshop. Descriptive analyses (i.e., mean and standard deviation) were used to describe participants’ characteristics.

### Step 3: content validity of the scale

The objective of this step was to validate the content of the PDDM rating scale.

#### Participants

Participants recruited from the content analysis step (see step 2) were enrolled.

#### Procedures and analysis

This third step, relating to content validity, focused on three criteria: (1) clinicians’ perception of the clarity of the item to avoid errors due to misunderstandings or misinterpretations, (2) clinicians’ satisfaction with the presentation of the item to be the most user-friendly and facilitate its use in clinical practice, and (3) clinicians’ perception of the clinical applicability of the item to determine its relevance for clinical practice and facilitate its integration in clinical practice.

During the analysis of the two clinical vignettes with the rating scale (see Step 2 procedure), the same participants answered the following three questions: 1) *Do these item statements seem clear to you?* 2) *Do these item statements appear to be presented satisfactorily?* and 3) *Do these item statements seem to be adapted to clinical practice?* These questions were answered with a 4-option Likert-type scale (1 = Not at all, 2 = A little, 3 = Mostly, and 4 = Totally).

The analysis was divided into two steps: i) statistical analyses to validate the five items, and ii) statistical analysis to validate the overall scale. For the first step, we used the Item level-Content Validity index (I-CVI) for each criterion [18]. I-CVI is defined as the number of participants rating the item either 3 or 4 divided by the total number of participants [19]. To determine if an item had to be revised or accepted, we used an I-CVI for each criterion [19]. If the item had to be revised, we used the feedback from the comments and suggestions section (See 2.2.2.) and we submitted the new version to the participants of the next workshop. According to the number of participants recruited for a workshop, we used different thresholds of the I-CVI to accept the item. If we recruited 4 participants or less, we applied a threshold of 1 to be accepted [20]. If we recruited between 5 and 10 participants, we applied a threshold of 0.78 to be accepted [18]. For each point estimate, we used a 95% confidence interval (95% CI) using the Wilson method. For the second step, after validating the content of each item, we used the Average-Content Validity Index (Ave-CVI) for each criterion to determine the clarity, presentation, and clinical applicability of the overall scale. We also used a global Ave-CVI corresponding to the mean of the Ave-CVIs for each criterion. This global Ave-CVI allowed us to appreciate the content validity of the overall scale. Ave-CVI corresponds to the average of the I-CVI values [19]. For each Ave-CVI, we applied a threshold of ≥0.9 [19], and we used a 95% confidence interval (95% CI) using the Wilson method. We used OpenEpi to obtain the 95%CI of each estimate.

### Decision rule to guide the process of content analysis and validity

**Fig. 1:**
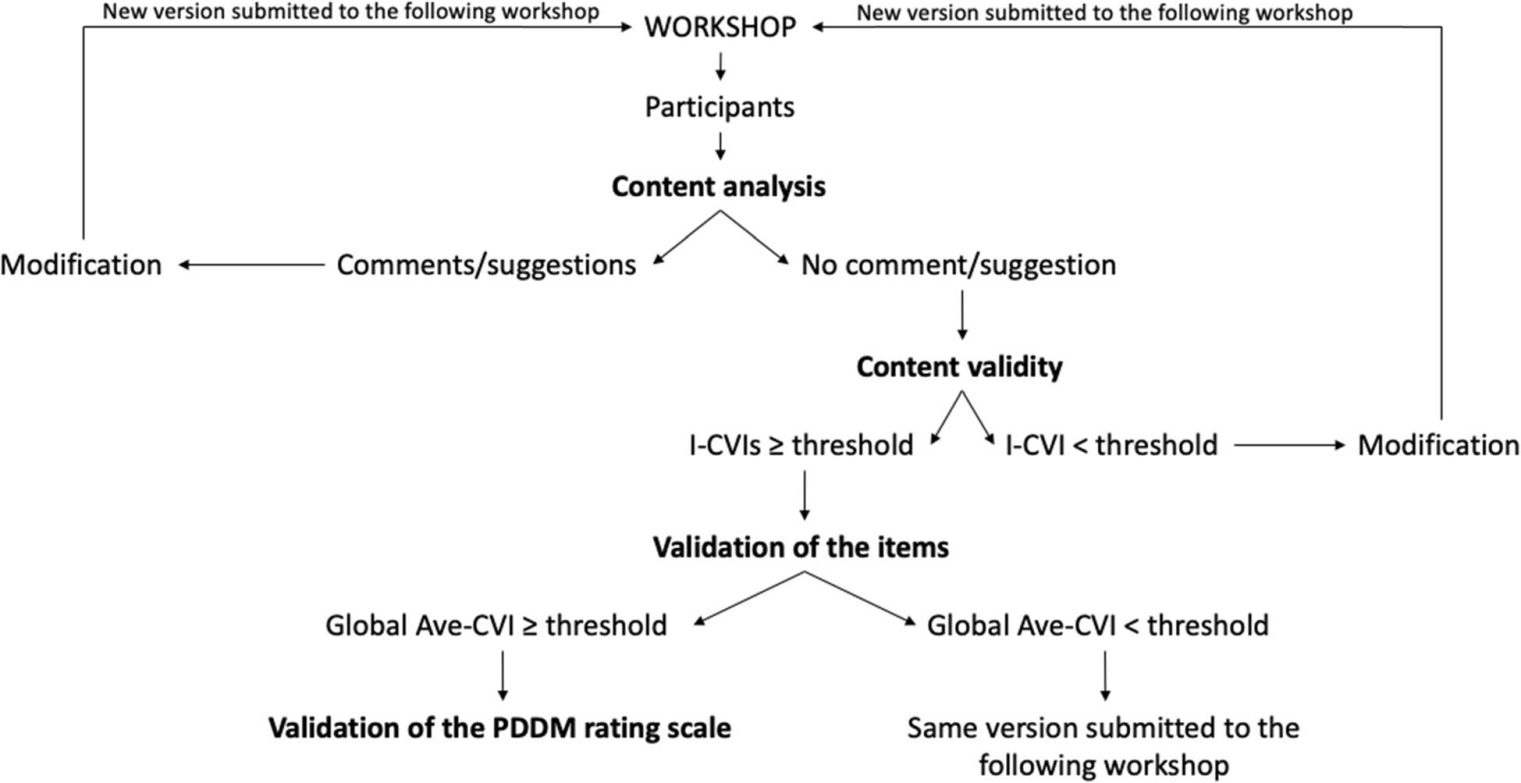
Decision rules for the content analysis and the content validity steps

The decision rule for the content analysis and content validity is illustrated in Fig. 1. To summarize it, the participants in workshop #1 provided feedback on the content of the rating scale. If these participants provided comments or suggestions, we modified the content of the scale and submitted the new version at the following workshop. We applied this iterative process until no further comments or suggestions were provided. We then proceeded to the content validity step during the same workshop where the participants rated the three criteria with the Likert-scale. If the I-CVI of each criterion was below the threshold (i.e., < 0.78 or 1 depending on the number of participants), we modified the content of the “problematic” items and submitted the new (updated) version to the following workshop and started over at the content analysis step. If the I-CVI of each criterion was above the threshold, the content of the items was validated. Then, we calculated the global Ave-CVI. If the global Ave-CVI was below the threshold (i.e., <.90), we submitted the rating scale to the following workshop. If the global Ave-CVI was above the threshold, the content of the PDDM rating scale was validated.

## Results

### Step 1: item generation to develop an initial rating scale

#### Operationalization of the rating scale

As the structure of the PDDM model is based on the separation of each domain into two complexity categories (categories A and B), “category screening” appeared to be the best solution to develop a rating scale to rapidly detect each domain’s contribution. For this “category screening”, we opted for the use of a threshold based on several present elements. Yet, in the absence of literature to support a given number, we deliberately decided to apply a low threshold [21] for screening and considered that the presence of at least one element within each category would suffice. For example, if the clinical assessment reveals the presence of one element within one category, the category is deemed “positive” and the clinician doesn’t have to systematically assess for the presence of other elements within the category.

Hence, we developed the following scoring method: For each domain/item, there are four possible options:

(A) Presence of at least one element from Category A,

(B) Presence of at least one element from Category B,

(A + B) Presence of at least one element from Categories A and B,

(0) Absence of elements in either A or B categories.

#### Item generation

The detailed results of this step are presented in Table 1. To avoid an overloaded scale, we created a mind map to support clinicians in the choice, use and interpretation of different questionnaires and procedures/instruments to screen the elements of each category (See Supplementary Material section). This mind map is available on https://pddmmodel.wordpress.com/.

**Table 1:** The 51 elements of the PDDM model and their concordance with the result of the comprehensive review

**Domain 1: Nociceptive pain drivers**		
Nominal elements included in each domain/category	Results from the items generation step	
Category A: Responders to LBP classification system	Instrument/tool or procedures (physical exam), or questions (anamnesis / self-reported)	Type of assessment
• *1/ Specific mechanical pattern*	• Treatment-Based Classification system (Alrwaily et al., 2016)^a^	PE
Category B: Non-responders to LBP classification system		
• *2/ LBP without any specific mechanical pattern*	• Treatment-Based Classification system (negative result)	PE
• *3/ Nociceptive pain related to identifiable structural stability deficit (post fracture, post- surgery)*	• Anamnesis for medical background/Tests for anatomical structures (Petersen et al., 2017)^a^	A/P
• *4/ Presence of signs/symptoms of an active inflammatory process*	• Specific signs and symptoms (InformedHealth.org (PubMed))^a^	A
**Domain 2: Nervous system dysfunction drivers**		
Category A: Peripheral sources of nervous system dysfunctions		
• *5/ Radicular pain pattern*	• Specific symptoms: anamnesis or self-reported (Mulvey et al., 2014)^a^	A
• *6/ Tingling/paresthesia or burning/shooting pain*	• Specific symptoms: anamnesis or self-reported (Mulvey et al., 2014)^a^	A
• *7/ Signs of radiculopathy*	• Specific symptoms: signs (van der Windt et al., 2010)^a^	A/PE
• *8/ Signs of myelopathy*	• Specific signs (Issack et al., 2012)^a^	PE
*Category B: Nervous system hypersensitivity*		
• *9/ Evidence of increased neural mechanosensitivity*	• Prone Knee Bend test (Alexander & Varacallo, 2021)^a^, Slump test (Urban & MacNeil, 2015)^a^, Straight Leg Raise test (Scaia et al., 2012)^a^	PE
• *10/ Evidence of hyperalgesia*	• Clinical signs and potential tests (Mücke et al., 2016)^a^	PE
• *11/ Evidence of allodynia*	• Clinical signs and potential tests (Mücke et al., 2016)^a^	PE
• *12/ Evidence of disproportionate pain intensity in relation to injury*	• Brief Pain Inventory (pain severity) (Poquet & Lin, 2016)^a^	Q
• *13/ Hypersensitivity of senses non-related to the MSK system*	• Central Sensitization Inventory-25 items (Q7–20) (Scerbo et al., 2017)^a^	Q
• *14/ Evidence of sympathetic nervous system dysfunctions*	• Specific signs (Liao et al., 2016)^a^	PE
• *15/ Symptoms of dysesthesia*	• Clinical signs and potential tests (Mücke et al., 2016)^a^	PE
• *16/ Evidence of widespread pain location*	• Central Sensitization Inventory-25 items (Q9) OR Central Sensitization Inventory-9 items (Q3) (Nishigami et al., 2018)^a^	Q
• *17/ Sleep disturbances secondary to painful symptoms*	• Central Sensitization Inventory-25 items (Q1–12–17-22) OR Central Sensitization Inventory-9 items (Q1–5) OR Brief Pain Inventory (Q9)	Q
**Domain 3: Comorbidity factors**		
Category A: Physical comorbidities		
• *18/ Co-occurring painful MSK pathologies (known/identified)* o *Osteoarthritis, rheumatoid arthritis, spondylarthritis, et* o *Any other painful MSK pathology triggering pain*	• Self-reported comorbidities (Hartvigsen et al., 2013)^a^	A
• *19/ Identified/known co-occurring disorders related to pain sensitization such as:* o *Chronic fatigue, migraines, irritable bowel syndrome, fibromyalgia*	• Self-reported comorbidities (Hestbaek et al., 2003; Rundell et al., 2017)^a^ OR Central Sensitization Inventory −25 items (Part B)	A/Q
Category B: Mental-health comorbidities		
• *20/ Mental health disorders (within the DSM-5)* o *Depressive disorders*	• Beck Depression Inventory-II (Harris & D’Eon, 2008)^a^	Q
o *Anxiety disorders*	• Central sensitization inventory −25 items (Q3–15) OR GAD-7 scale (Plummer et al., 2016)^a^	Q
o *Personality disorders*	• Standardised Assessment of Personnality – Abbreviated scale (Germans et al., 2012)^a^	Q
o *History of substance-use disorder*	• Anamnesis	A
• *21/ Post-traumatic stress disorders (PTSD)*	• Post-Traumatic Stress Disorder-8 Scale (Andersen et al., 2017)^a^	Q
• *22/ Sleep disorders*	• Central Sensitization Inventory −25 items (part B) OR (Insomnia Severity Index (Alsaadi et al., 2013)^a^ AND/OR Fatigue Severity Scale (Takasaki & Treleaven, 2013)^a^)	Q
**Domain 4: Cognitive-emotional drivers**		
Category A: Maladaptive cognitions and emotions		
	• STart Back Screening Tool: This tool does not cover element of this category. However, its prognostic capacity (prediction of disability at 6 months) based on psychosocial factors (mainly cognitive-emotional) is relevant for clinicians (Beneciuk 2013, Hill 2008)^a^	Q
• *23/ Pain catastrophizing*	• Pain Catastrophizing Scale (Osman 2000)^a^	Q
• *24/ Pain-related anxiety*	• Pain Anxiety Symptoms Scale-20 (Coons 2004)^a^	Q
• *25/ Negative mood*	• Central Sensitization Inventory-25 items (Q16) OR Beck Depression Inventory-II	Q
• *26/ Fear of movement / kinesiophobia*	• Tampa Scale of Kinesiophobia-17 items (Roelofs 2011)^a^	Q
• *27/ Pain-related fears*	• Fear Avoidance Components Scale (Neblett 2016)^a^	Q
• *28/ Poor self-efficacity*	• Chronic Disease Self-Efficacy Scales (Brady 2011)^a^	Q
• *29/ High illness perception*	• Brief Illness Perception Questionnaire (Hallegraef 2013)^a^	Q
• *30/ Pain expectations*	• Brief Illness Perception Questionnaire	Q
• *31/ Negative/low expectation of recovery*	• Brief Illness Perception Questionnaire	Q
• *32/ Low pain coping*	• Chronic Pain Coping Inventory (Jensen 2003)^a^	Q
• *33/ Poor knowledge relating to pain science*	• Revised Neurophysiology of Pain (Catley 2013)^a^ OR Fear Avoidance Beliefs Questionnaire (Swinkles 2003)^a^	Q
• *34/ Perceived injustice*	• Injustice Experience Questionnaire (Sullivan 2008)^a^	Q
• *35/ Perception that medical treatments are still necessary or uncomplete*	• Brief Illness Perception Questionnaire	Q
Category B: Maladaptive pain behaviors		
• *36/ Facial expressions*	• List of observable pain behaviors (Naye 2021)^a^	A/PE
• *37/ Verbal/paraverbal pain expressions*	• If clinicians want a quantified assessment of this category:	
• *38/ Guarded postures*	o Avoidance behaviors: BAT-Back (Holzapfel 2016)^a^	PE
• *39/ Bending/rubbing the back after performing an activity*	o Endurance behaviors: Avoidance Endurance Questionnaire (Hasenbring 2009)^a^	Q
• *40/ Completely avoiding to perform a task*		
• *41/ Discordance between reported behaviors (by the patient) and observed behaviors (by the therapist)*		
**Domain 5: Contextual drivers**		
Category A: Occupational context		
	• Örebro Musculoskeletal Pain Screening Questionnaire-short form: This tool does not cover element of this category. However, its prognostic capacity (prediction of return to work at 6 months) based on psychosocial factors are relevant for clinicians (Fuhro 2016).	Q
• *42/ Low return-to-work expectations*	• Anamnesis OR Obstacles to Return-to-Work Questionnaire (Part 3) (Marhold 2002)^a^	A/Q
• *43/ Low job satisfaction*	• Anamnesis OR Obstacles to Return-to-Work Questionnaire (Part 2)	A/Q
• *44/ Perception of heavy work*	• Anamnesis OR Obstacles to Return-to-Work Questionnaire (Part 3)	A/Q
• *45/ High job stress*	• Anamnesis OR Obstacles to Return-to-Work Questionnaire (Part 3)	A/Q
• *46/ High occupational demands*	• Anamnesis OR Obstacles to Return-to-Work Questionnaire (Part 3)	A/Q
• *47/ Low job flexibility*	• Anamnesis OR Obstacles to Return-to-Work Questionnaire (Part 3)	A/Q
• *48/ Employer’s policies regarding return-to-work are limited or restrictive*	• Anamnesis OR Obstacles to Return-to-Work Questionnaire (Part 3)	A/Q
Category B: Social context		
• *49/ Poor attitudes of employer, family or health care professionals*	• Anamnesis	A
• *50/ Low or non-access to care*	• Anamnesis	A
• *51/ Communication barriers*	• Anamnesis	A

*LBP* Low back pain, *MSK* Musculoskeletal

**A:** information collected by anamnesis or self-reported (subjective exam), **PE:** Physical examination (requires specific procedures), **Q:** Information collected by questionnaire or measurement tools

^a^The detailed references are available in Supplementary Material section

The initial version of the PDDM rating scale is presented in Supplementary Material section.

### Step 2: content analysis to refine the scale

**Fig. 2:**
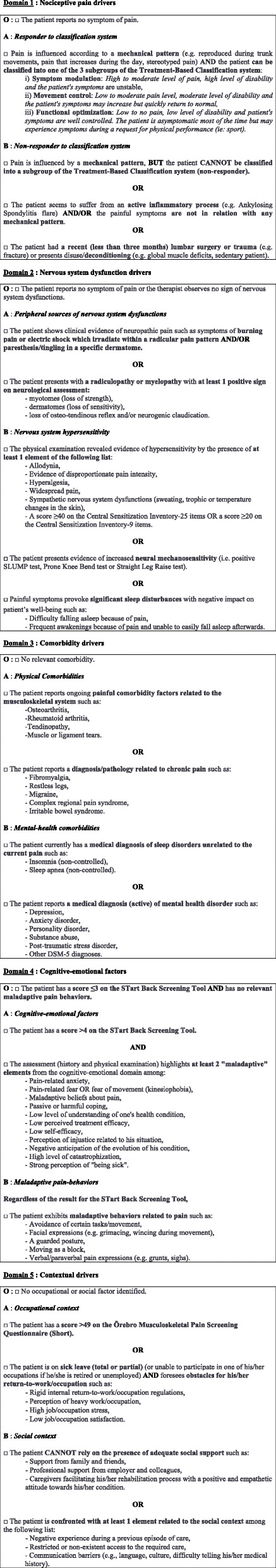
Final version of the PDDM rating scale

We needed 3 workshops to obtain a result of no comments or suggestions on the content of the rating scale. Over the 3 workshops (2 in person and 1 online due to the COVID-19 pandemic) we were able to recruit 42 participants, with no prior exposure to the PDDM, with a mean of 17,6 years of experience (±12,4). Fifteen participants (35,7%) previously received training on a classification system and 27 (64,3%) rarely to always used questionnaires in their daily clinical practice. In the first workshop, 14 participants shared their perception of and satisfaction with the rating scale. Five participants suggested modifying the presentation of Category A of domain #5 (contextual drivers) to highlight the fact that the patient “perceives obstacles to returning to work”. We made this modification, and the updated version was presented during the following workshop. In the second workshop, 12 participants were recruited. For domain #1 (nociceptive pain drivers), 3 participants reported the fact that they needed more information to facilitate the integration of the classification system. We therefore integrated the main physical characteristics of the 3 subgroups of the Treatment-Based Classification into the rating scale. For the second domain (nervous system dysfunction drivers),1 participant reported the need for examples of sleep disturbances. We added this information to the rating scale. For the third domain (comorbidity drivers), 3 participants asked whether they had to consider a stabilized or past comorbidity. We modified the item by adding “non-controlled” for mental-health and sleep disorders. For the fourth domain (cognitive-emotional drivers), 2 participants asked if the STart Back Screening Tool had to be > 3 for Category B. We modified the item by adding “Regardless of the result of the STart Back Screening Tool, check if the patient has developed maladaptive pain behaviors”. In a more general perspective, a participant highlighted the fact that the different item presentations were not homogenous. We therefore modified the items to facilitate understanding and to make it easier to detect the key characteristics of each category. This new version was tested with 16 participants during the third workshop. No comments were made. At the end of this step, we obtained a rating scale refined by primary users, and ready to be validated (Fig. 2).

### Step 3: content validity of the rating scale

As the participants of the third workshop (*n* = 16) did not make comments or suggestions, we collected data to perform the content validity analysis during this 3rd workshop.

#### Items validation

The number of participants in the 3rd workshop (*n* = 16) allowed us to apply the I-CVI threshold of 0.78. The I-CVIs of the clarity, presentation, and clinical applicability of the item were above the threshold (Table 2). Certain lower bounds of the confidence interval were below the threshold. Thus, the content of the five items was validated. According to our decision tree (Fig. 1), we were able to continue the process with the validation of the overall scale.

**Table 2:** Results of the content validity analyses (step 3)

	3rd workshop (*n* = 16)
**Domain 1**	
*Clarity of the item* I-CVI = 95%CI	1 [0.81; 1]
*Presentation of the item* I-CVI = 95%CI	0.94 [0.72; 0.99]
*Clinical applicability of the item* I-CVI = 95%CI	0.94 [0.72; 0.99]
**Domain 2**	
*Clarity of the item I*-CVI = 95%CI	0.94 [0.72; 0.99]
*Presentation of the item I*-CVI = 95%CI	1 [0.81; 1]
*Clinical applicability of the item* I-CVI = 95%CI	0.94 [0.72; 0.99]
**Domain 3**	
*Clarity of the item* I-CVI = 95%CI	1 [0.81; 1]
*Presentation of the item* I-CVI = 95%CI	1 [0.81; 1]
*Clinical applicability of the item* I-CVI = 95%CI	0.94 [0.72; 0.99]
**Domain 4**	
*Clarity of the item* I-CVI = 95%CI	0.94 [0.72; 0.99]
*Presentation of the item* I-CVI = 95%CI	1 [0.81; 1]
*Clinical applicability of the item* I-CVI = 95%CI	0.94 [0.72; 0.99]
**Domain 5**	
*Clarity of the item* I-CVI = 95%CI	0.94 [0.72; 0.99]
*Presentation of the item* I-CVI = 95%CI	1 [0.81; 1]
*Clinical applicability of the item* I-CVI = 95%CI	0.94 [0.72; 0.99]
**Overall scale**	
Ave-CVI for the clarity of the scale 95% CI	0.96 [0.90; 0.99]
Ave-CVI for the presentation of the scale 95% CI	0.99 [0.93; 1]
Ave-CVI for the clinical applicability of the scale 95% CI	0.94 [0.86; 0.97]
Global Ave-CVI (Ave-CVI for the overall scale) 95% CI	0.96 [0.93; 0.98]

*I-CVI* Item level-Content Validity Index = Number of participants rating the item either 3 or 4 / Total number of participants. I-CVI threshold: 0.78

*Ave-CVI* Average-Content Validity Index = Average of the I-CVI values. Ave-CVI threshold: 0.9

#### Scale validation

The Ave-CVI for the clarity of the scale was 0.96 [0.9;0.99], the Ave-CVI for the presentation of the scale was 0.99 [0.93;1], and the Ave-CVI for the clinical applicability of the scale was 0.94 [0.86;0.97] (Table 2). All these Ave-CVI were above the threshold of 0.9, but the lower bound for the clinical applicability was below it. Concerning the overall scale, the Ave-CVI was 0.96 [0.93;0.98] and, was above the threshold (see Table 2).

## Discussion

From the original PDDM model and a comprehensive review, we developed a rating scale which allows to detect the contribution of each domain to establish the patient’s biopsychosocial profile. Clinicians participating in workshops on the PDDM model provided feedback that allowed us to refine the scale. We validated the content of the PDDM rating scale using content validity index at item (I-CVI) and overall scale (Ave-CVI) levels. To our knowledge, this rating scale is the first to be developed based on a theoretical diagnostic framework for people with low back pain. Our study led to three main observations.

The development of a biopsychosocial tool to establish a profile, such as our rating scale, requires the incorporation of multiple concepts. Knowledge of these concepts is considered by physiotherapists to be an important barrier to the integration of a biopsychosocial perspective [10–13, 15]. We also know that integration of a biopsychosocial perspective is more difficult when physiotherapists need to change their practice [11, 13]. The fact that we collected feedback from a broad range of physiotherapist backgrounds allowed us to refine the rating scale by incorporating more information to facilitate its understanding by future users as well as the concepts that need more information during the workshop. However, our recruitment strategy led to two limitations: First, a more specialized sample with expertise on biopsychosocial approaches might be more helpful to critique the instruments or procedures included in the rating scale. Second, after a thorough workshop on the contribution of a biopsychosocial perspective in rehabilitation care, social desirability bias could impact participants’ rating or comments [22].

For feasibility considerations, we collected the data towards the end of the workshop. Consequently, participants were “tested” without a familiarization period. Thus, we could not collect information on the ease of use, clinical utility, and clinical relevance of the rating scale. These feasibility considerations led us to use clinical vignettes (versus real patients) to gather feedback. With clinical vignettes, participants mainly used their clinical reasoning skills rather than their “true” abilities to collect data [23]. Moreover, clinical vignettes did not allow participants to complete their assessment with their own clinical reasoning process and prevented communication to further assess certain elements. From the perspective of the knowledge-to-action framework [24], collecting feedback from participants with a PDDM model exposition in their daily clinical practice could be extremely useful; thus further studies are required.

Detecting the contribution of each domain is an important step in applying a biopsychosocial approach with the PDDM model. Guided by the contribution of each domain (or combination of domains), physiotherapists can tailor their treatment plan according to the patient’s profile. The development of the PDDM rating scale opens the door to the proposals of recommended interventions, based on the patient’s profile. These treatment proposals are one of the needs highlighted by participants in our feasibility trial [9]. Establishing a profile could also help clinicians modify the patient’s biomedical beliefs and expectations [25–28].

### Limitations

The main limitation concerning the use of the Content Validity Index is its inflation of agreement due to chance [29]. However, according to Polit et al. [18], an I-CVI threshold of 0.78 is sufficient to obtain a good to excellent modified kappa, regardless of the number of participants. Some of the lower bounds of the I-CVI confidence interval, as well as the confidence interval lower bound of the Ave-CVI for clinical applicability of the overall scale were below the threshold, we therefore must be cautious when interpreting our results. However, with the small sample size needed to perform CVI analyses, the confidence intervals are inevitably large. But the use of the 95% confidence interval allowed us to apply a conservative approach in interpreting the results. Also, the lower bound of the global Ave-CVI confidence interval was above the threshold, which makes it possible to conclude on the overall scale’s content validity.

This scale’s development depended on clinical and scientific constraints. Sub-optimal choices had to be made to limit clinical constraints. Actual evidence led us to choose a dichotomous screening of categories rather than a weighted contribution that could give more information to guide clinicians in the prioritization of care. Although essential, this content validity step is not enough to conclude on the validity of the scale [30]. We must continue the psychometric properties process and determine the real clinical utility of this rating scale in treatment decision making.

## Conclusion

We developed a 5-item rating scale that allows clinicians to rapidly detect the contribution of each of the PDDM model’s domains. This screening allows to establish the patient’s biopsychosocial profile. The content of the scale was first refined by a sample of clinicians with no prior exposure to the PDDM model and who attended a 1-day workshop on the model. All the I-CVI and Ave-CVI results were above the recommended thresholds. These statistical analyses allowed us to validate the content of the developed rating scale with a good level of quality evidence. Future steps are required to continue the psychometric properties process of this rating scale.

## Supplementary Information

**Additional file 1.**

**Additional file 2.**

## Data Availability

The datasets used and/or analysed during the current study are available from the corresponding author on reasonable request.

## Abbreviations

Ave-CVI: Average-Content Validity Index
COSMIN: COnsensus-based Standards for the selection of health Measurement INstruments
I-CVI: Item level-Content Validity Index
LBP: Low Back Pain
PDDM: Pain and Disability Drivers Management
95%CI: 95% Confidence Interval
