# Supplementary material for: Development and content validity of a rating scale for the pain and disability drivers management model
Source: Arch Physiother. 2022 May 16;12:14. doi: 10.1186/s40945-022-00137-2 (PMC9107946; doi:10.1186/s40945-022-00137-2)
Supplement: Supplementary file 1 — Additional file 1. [file 40945_2022_137_MOESM1_ESM.docx]

**Supplementary Material 1: Scale development**

**METHODS**

*Search strategy*

To respond to this scale objective (i.e., rapidly establish the presence or absence of the categories of each domain of the model), we used a 2-step search strategy:

1/ To determine if one or more screening tool(s) are existing to detect the presence of the entire category. For example, does a screening tool regarding nervous system hypersensitivity exist? If the screening tool covers all the elements of this category, step 2 is not considered.

2/ If a screening tool does not exist or if they partially integrate the elements of the category, the second step allows determining if one or more assessment tool(s) for each non-covered element exist.

Thus, for the first step, the search equation includes: a/ **target population** ((« low back pain »[Title] OR « musculoskeletal pain »[Title] OR « musculoskeletal condition* »[Title] OR « chronic pain »[Title]), b/ **the category of the model and its synonyms** (for example, « pain behavior* »[Title] OR « pain behaviour* »[Title] OR « communicative behavior* »[Title] OR « communicative behaviour* »[Title] OR « protective behavior* »[Title] OR « protective behaviour* »[Title] OR avoidance[Title] OR endurance[Title]), and c/ **screening** (« screen* »[Title]). For this strategy, the [Title] filter can involve toward [Title/Abstract] filter if the initial equation does not allow obtaining satisfying results.

For the second step, the search equation includes: a/ **the target population**, b/ **the element of the category and its synonyms** (for example, « sensory change* »[Title] OR allodyni*[Title] OR hyperalgesi*[Title] OR hypoesthesi*[Title] OR hypesthesi*[Title] OR hyperesthesi*[Title] paresthesi*[Title]), c/ **assessment and its synonyms as well as assessment-related words** (assess*[Title] OR assessment[Title] OR evaluat*[Title] OR measure*[Title] OR exam*[Title] OR diagnos*[Title] OR questionnaire*[Title] OR scale*[Title] OR index[Title] OR inventory[Title] OR tool*[Title]). For this strategy, the [Title] filter can involve toward [Title/Abstract] filter if the initial equation does not allow obtaining satisfying results.

We applied this search strategy in two databases: PubMed and Scopus.

*Eligibility principles and criteria*

We applied six clinical principles for selecting the measure instruments/procedures: 1/ Time to complete the instrument/procedure, 2/ the need of equipment to perform the procedure and its cost, 3/ the clinical utility (for example, if a 5-item questionnaire brings the same information as a 30 min test, we will prioritize the questionnaire), 4/ the clinical habits regarding procedures (for example, a known and well-disseminated procedure such as neurological exam will be prioritized beside a recent and few disseminated procedure), 5/ clinicians, knowledge regarding the measured concept (for example, an unfamiliar concept such as central sensitization would require the selection of several instruments/procedures in order to give clinicians the choice to use the procedure that best suits them), and 6/ Psychometric data comparison.

We also applied a selection criterion: i/ the instrument or procedure must be validated for low back pain or chronic pain.

*Selection process*

An evaluator (FN) performed the selection process according to the six principles and the inclusion criterion. After the application of the search strategy, the evaluator made a list of the instruments/procedures validated for our target population (inclusion criterion) and able to screen the category of interest (or assess the element of interest). Then, for the clinical principles (principles 1 to 5), the evaluator extracted available data from the development study of the instrument/procedure. If this data was not available in this study, the evaluator performed a targeted research on PubMed. Finally, for the principle regarding psychometric properties, the evaluator privileged data from systematic review or psychometric meta-analysis. If synthesis review was not available, the evaluator performed a targeted research on PubMed to find all articles in relation with validity and reliability of the instruments/procedures.

The finale decision regarding the instruments/procedures included in the rating scale was made in consultation with a second reviewer (YTL).

**RESULTS**

Briefly, we found screening and/or assessment instruments/procedures that allow rapidly screening the categories of the five domains excepted for the category of maladaptive pain behaviors. The Delphi method has validated observable elements for this category. Thus, we performed a systematic search and review in order to determine the observable pain behaviors that patients can exhibit during rehabilitation (1). The Table 1 allows matching the elements of the model with the chosen instruments/procedures.

In order to make the selection process explicit, the following section presents the example of the category of the nervous system hypersensitivity. The search equation was: (("low back pain"[Title] OR "musculoskeletal pain"[Title] OR "musculoskeletal condition*"[Title] OR "chronic pain"[Title]) AND ("nervous system hypersensitivity"[Title] OR "central sensitization"[Title]) AND screen*[Title]). No screening instrument/procedure was found. We modified the filter of screening word from [Title] to [Title/Abstract]. This strategy allowed finding four studies on PubMed and five studies on Scopus regarding the screening of nervous system hypersensitivity. The first step of the selection process was to elaborate a list of the available instruments/procedures. Three screening instruments/procedures were validated for our population: the Central Sensitization Inventory-25 items, the Central Sensitization-9 items, and the Quantitative Sensory Testing. The Table 2 synthesized the selection principles. At the end of the selection process, we retained the Central Sensitization Inventory-25 items (CSI-25). However, CSI-9 has the advantage of being faster than the CSI-25, we opted to incorporate it despite the data source (data source of CSI-25: systematic review, data source of CSI-9: development and psychometric properties study). Also, these questionnaires did not cover the entirety of the elements included in the category B of the nervous system dysfunction drivers domain. Thus, we performed search equations for the remaining elements in order to determine assessment instruments/procedures of which explanation, cut-off and interpretation are available in a mind map on the PDDM model (See the following section). Finally, we built the item with three distinct parts: 1/ central sensitization-related elements, 2/ the mechanosensitivity (because the mechanosensitivity can be positive without central sensitization), and 3/ sleep disturbances (because they can be present before central sensitization (2).

**Help with the use of the scale**

The developed rating scale is composed of numerous recent concepts (e.g., central sensitization), unfamiliar (e.g., self-efficacy), and assessed or screened with few disseminated instruments/procedures. In order to facilitate the clinical integration of this rating scale without overloading the content of the scale, we decided to develop a website with a mind map of the PDDM model (Figure 1). This mind map proposes the clinical reasoning associated with the model, the structure and content of the model, and also pedagogic content. For each element, presentation of the concept and an assessment tool with its interpretation are available (Figure 2). Thus, clinicians can familiarize with the model. This mind map allows detailing the content of the developed rating scale. For example, the mind map details the signs of an active inflammatory process mentioned in the category B of the nervous system dysfunction drivers domain of the scale (Figure 3).

**Table 1:** Match between the elements of the PDDM model and the instruments/procedures included in the rating scale.

| Domain 1: NOCICEPTIVE PAIN DRIVERS | |
| --- | --- |
| *Category A: Responder to Classification system* | *Chosen instruments/procedures* |
| - *1/ Specific mechanical pattern of pain* | - Treatment-Based Classification (3) |
| *Category B: Non-responder to classification system* |  |
| - *2/ Low back pain without any specific mechanical pattern* - *3/ Nociceptive pain related to identifiable structural stability deficits (post-fracture, post-surgery)* - *4/ Presence of signs/symptoms of an active inflammatory process* | - Treatment-Based Classification (negative result) - Anamnesis for medical history/Procedures from Petersen et al. for anatomic structures (4) - Signs and symptoms from InformedHealth.org (PubMed) |
| Domain 2: NERVOUS SYSTEM DYSFUNCTION DRIVERS | |
| *Category A: Peripheral sources of nervous system dysfunctions* |  |
| - *5/ Radicular pain pattern* - *6/ Tingling/paresthesia or burning/shooting pain* - *7/ Signs of radiculopathy* - *8/ Signs of myelopathy* | - Anamnesis or self-reported from Mulvey et al. (5) - Anamnesis or self-reported from Mulvey et al. (5) - Signs from van der Windt et al. (6) - Signs from Issack et al. (7) |
| *Category B: Nervous system hypersensitivity* |  |
| - *9/ Evidence of increased neural mechanosensitivity* - *10/ Evidence of hyperalgesia* - *11/ Evidence of allodynia* - *12/ Evidence of disproportionate pain intensity in relation to injury* - *13/ Hypersensitivity of senses unrelated to the MSK system* - *14/ Evidence of sympathetic nervous system dysfunction (i.e. sweating/dryness, skin temperature changes)* - *15/ Symptoms of dysesthesia* - *16/ Evidence of widespread pain location* - *17/ Sleep disturbances secondary to painful symptoms* | - Prone knee bend test (8), Slump test (9), Straight Leg Raise test (10) - Clinical signs and possible tests from Mücke et al. (11) - Clinical signs and possible tests from Mücke et al. (11) - Brief Pain Inventory (pain severity) (12) - Central Sensitization Inventory-25 items (Q7-20) (13) - Signs from Liao et al. (14) - Clinical signs and possible tests from Mücke et al. (11) - Central Sensitization Inventory-25 items (Q9) OR CSI-9 (Q3) (15) - Central Sensitization Inventory-25 items (Q1-12-17-22) OR CSI-9 (Q1-5) OR Brief Pain Inventory (Q9) |
| Domain 3: COMORBIDITY DRIVERS | |
| *Category A: Physical comorbidities* |  |
| - *18/ Identified/known co-occurring painful musculoskeletal pathologies*   - *Osteoarthritis, Rheumatoid arthritis, Spondylarthritis, etc*   - *Any other painful musculoskeletal pathology triggering pain* - *19/ Identified/known co-occurring disorders related to pain sensitization, such as:*    - *Chronic fatigue, Migraines, Irritable bowel syndrome, Fibromyalgia* | - Comorbidities from Hartvigsen et al. (16) - Comorbidities from Rundell et al. (17), Hestbaeck et al. (18), OR Central Sensitization Inventory-25 items (Part B) |
| *Category B: Mental-health comorbidities* |  |
| - *20/ Mental health disorders (within the DSM-5):*   - *Depressive disorders*   - *Anxiety disorders*   - *Personality disorders*   - *History of Substance-use disorder*   - *21/ PTSD (Post-Traumatic Stress Disorder)* - *22/ Patient-reported sleep disorders* | - Beck Depression Inventory-II (19) - Central Sensitization Inventory-25 items (Q3-15) OR Generalized Anxiety Dirsorder-7 (20) - Standardised Assessment of Personnality – Abbreviated scale (21) - Anamnesis - Post-Traumatic Stress Disorder-8 Scale (22) - Central Sensitization Inventory (Part B) OR (Insomnia Severity Index (23) AND/OR Fatigue Severity Scale (24)) |
| Domain 4: COGNITIVE-EMOTIONAL DRIVERS | |
| *Category A: Maladaptive cognitive-emotional factors* |  |
| - *23/ Pain catastrophizing* - *24/ Pain-related anxiety* - *25/ Negative mood* - *26/ Fear of movement* - *27/ Pain-related fears* - *28/ Poor Self-efficacy* - *29/ High Illness perception* - *30/ Pain expectations* - *31/ Negative/low expectation of recovery* - *32/ Low Pain coping* - *33/ Poor knowledge relating to pain science* - *34/ Perceived injustice* - *35/ Perception that medical treatments are still needed or incomplete* | - STart Back Screening tool: The tool covers no element of this category. However, its prognostic ability (prediction of disability) based on psychosocial factors is relevant for clinicians (25,26). - Pain Catastrophizing Scale (27) - Pain Anxiety Symptoms Scale-20 (28) - Central Sensitization Inventory-25 items (Q16) OR Beck Depression Inventory-II - Tampa Scale of Kinesiophobia-17 items (29) - Fear Avoidance Components Scale (30) - Chronic Pain Self Efficacy Scale (31) - Brief Illness Perception Questionnaire (32) - Brief Illness Perception Questionnaire - Brief Illness Perception Questionnaire - Chronic Pain Coping Inventory (33) - Revised neurophysiology of pain (34) OR Fear Avoidance Beliefs Questionnaire (35) - Injustice Experience Questionnaire (36) - Brief Illness Perception Questionnaire |
| *Category B: Maladaptive pain behaviors* |  |
| - *36/ Facial expressions (e.g., grimacing or wincing)* - *37/ Verbal/paraverbal pain expressions (e.g., pain words, grunts, sighs, and moans)* - *38/ A guarded posture (e.g., keeping the back straight while lifting)* - *39/ Bending/rubbing the back after performing an activity* - *40/ Completely avoiding performing a task* - *41/ Discordance between reported behaviors (by the patient) and observed behaviors (by the therapist)* | - List of observable pain behaviors (1) - If clinicians want to quantify this category, the mind map proposes:   - *Avoidance behaviors: BAT-Back (37)*   - *Endurance behaviors: Avoidance Endurance Questionnaire (38)* |
| Domain 5: CONTEXTUAL DRIVERS | |
| *Category A: Occupational context* |  |
| - *42/ Low return to work expectations* - *43/ Low Job satisfaction* - *44/ Perception of heavy work* - *45/ High job stress* - *46/ High Occupational demands* - *47/ Job flexibility* - *48/ Employer’s policies regarding return to work are limited or restrictive* | - Örebro Musculoskeletal Pain Screening Questionnaire-short form: The tool covers no element of this category. However, its prognostic ability (prediction of return-to-work) based on psychosocial factors is relevant for clinicians (39) - Anamnesis OR Obstacles to Return-to-Work Questionnaire (part 3) (40) - Anamnesis OR Obstacles to Return-to-Work Questionnaire (part 2) - Anamnesis OR Obstacles to Return-to-Work Questionnaire (part 2) - Anamnesis OR Obstacles to Return-to-Work Questionnaire (part 2) - Anamnesis OR Obstacles to Return-to-Work Questionnaire (part 2) - Anamnesis OR Obstacles to Return-to-Work Questionnaire (part 2) - Anamnesis OR Obstacles to Return-to-Work Questionnaire (part 2) |
| *Category B: Social context* |  |
| - *49/ Poor attitudes of employer, family or health care professionals* - *50/ Low or non-access to care* - *51/ Communication barriers* | - Anamnesis - Anamnesis - Anamnesis |

**Table 2:** Selection principles for the screening tool of the category B of the nervous system hypersensitivity drivers domain.

| **Tool** | **Time to complete** | **Equipment** | **Clinical utility** | **Clinical habits** | **Clinicians’ knowledge** | **Psychometric properties** | **Data from** |
| --- | --- | --- | --- | --- | --- | --- | --- |
| Central Sensitization Inventory-25 items (13,41) | No information but presume <10 min | No specific equipment required | ·Cut-off  ·Severity levels | No well-disseminated | No well known | ·Reliable  ·Valid | Systematic review |
| Central Sensitization Inventory-9 items (15,42) | No information but presume <10 min | No specific equipment required | Cut-off | No well-disseminated | No well known | ·Reliable  ·Valid | Development study |
| Quantitative Sensory Testing (43) | Time-consuming | ·Specific equipment required  ·Expensive | Cut-off | No well-disseminated | No well known | Reliable | Systematic review and meta-analysis |

**Figure 1:** The developed mind map of the PDDM model.

**Figure 2:** PDDM mind map: Example of the branch related to nervous system dysfunction drivers domain.


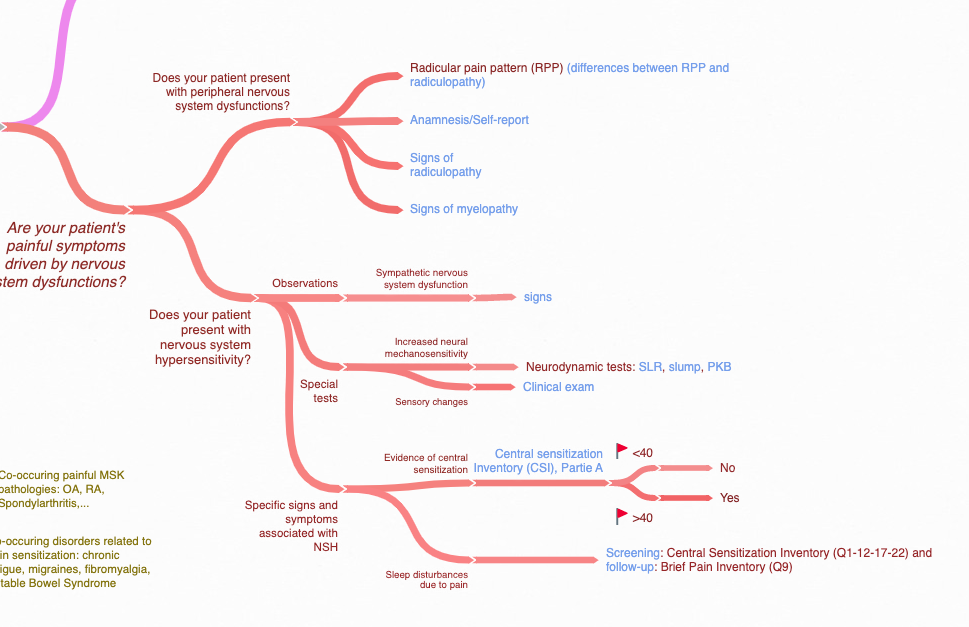


**Figure 3:** Pedagogic content included in the mind map. Example for signs of radiculopathy and interpretation of the Central Sensitization Inventory.


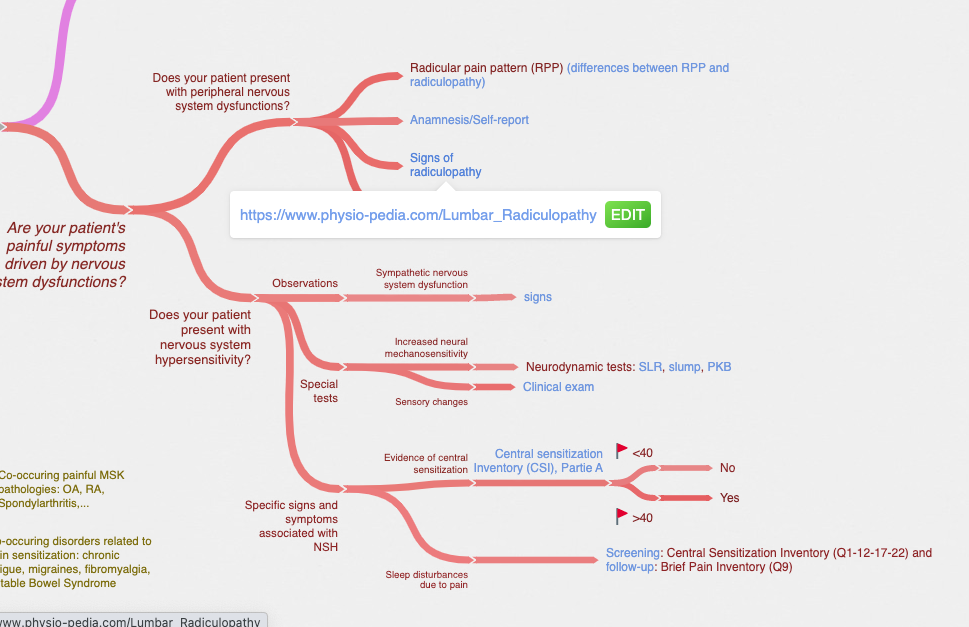


1. Naye F, Cachinho C, Tremblay A-P, Saint-Germain Lavoie M, Lepage G, Larochelle E, et al. How to objectively assess and observe maladaptive pain behaviors in clinical rehabilitation: a systematic search and review. Arch Physiother. 2021 Jun 3;11(1):15.

2. Iacovides S, George K, Kamerman P, Baker FC. Sleep Fragmentation Hypersensitizes Healthy Young Women to Deep and Superficial Experimental Pain. J Pain Off J Am Pain Soc. 2017;18(7):844–54.

3. Alrwaily M, Timko M, Schneider M, Stevans J, Bise C, Hariharan K, et al. Treatment-Based Classification System for Low Back Pain: Revision and Update. Phys Ther. 2016 Jun;96(7):1057 LP – 1066.

4. Petersen T, Laslett M, Juhl C. Clinical classification in low back pain: best-evidence diagnostic rules based on systematic reviews. BMC Musculoskelet Disord [Internet]. 2017 May 12 [cited 2017 Oct 28];18. Available from: https://www.ncbi.nlm.nih.gov/pmc/articles/PMC5429540/

5. Mulvey MR, Bennett MI, Liwowsky I, Freynhagen R. The role of screening tools in diagnosing neuropathic pain. Pain Manag. 2014 May;4(3):233–43.

6. van der Windt DA, Simons E, Riphagen II, Ammendolia C, Verhagen AP, Laslett M, et al. Physical examination for lumbar radiculopathy due to disc herniation in patients with low‐back pain. In: The Cochrane Library [Internet]. John Wiley & Sons, Ltd; 2010 [cited 2018 May 23]. Available from: http://cochranelibrary-wiley.com/doi/10.1002/14651858.CD007431.pub2/full

7. Issack PS, Cunningham ME, Pumberger M, Hughes AP, Cammisa FP. Degenerative lumbar spinal stenosis: evaluation and management. J Am Acad Orthop Surg. 2012 Aug;20(8):527–35.

8. Alexander CE, Varacallo M. Lumbosacral Radiculopathy. In: StatPearls [Internet]. Treasure Island (FL): StatPearls Publishing; 2021 [cited 2021 Aug 3]. Available from: http://www.ncbi.nlm.nih.gov/books/NBK430837/

9. Urban LM, MacNeil BJ. Diagnostic Accuracy of the Slump Test for Identifying Neuropathic Pain in the Lower Limb. J Orthop Sports Phys Ther. 2015 Aug;45(8):596–603.

10. Scaia V, Baxter D, Cook C. The pain provocation-based straight leg raise test for diagnosis of lumbar disc herniation, lumbar radiculopathy, and/or sciatica: A systematic review of clinical utility. J Back Musculoskelet Rehabil. 2012 Jan 1;25(4):215–23.

11. Mücke M, Cuhls H, Radbruch L, Baron R, Maier C, Tölle T, et al. Quantitative sensory testing (QST). English version. Schmerz Berl Ger. 2016 Jan 29;

12. Poquet N, Lin C. The Brief Pain Inventory (BPI). J Physiother. 2016 Jan;62(1):52.

13. Scerbo T, Colasurdo J, Dunn S, Unger J, Nijs J, Cook C. Measurement Properties of the Central Sensitization Inventory: A Systematic Review. Pain Pract Off J World Inst Pain. 2017 Aug 29;

14. Liao CD, Tsauo JY, Liou T-H, Chen H-C, Rau CL. Efficacy of noninvasive stellate ganglion blockade performed using physical agent modalities in patients with sympathetic hyperactivity-associated disorders: A systematic review and meta-analysis. PLoS One [Internet]. 2016 Dec 1 [cited 2018 May 17];11(12). Available from: https://tmu.pure.elsevier.com/en/publications/efficacy-of-noninvasive-stellate-ganglion-blockade-performed-usin

15. Nishigami T, Tanaka K, Mibu A, Manfuku M, Yono S, Tanabe A. Development and psychometric properties of short form of central sensitization inventory in participants with musculoskeletal pain: A cross-sectional study. PloS One. 2018;13(7):e0200152.

16. Hartvigsen J, Natvig B, Ferreira M. Is it all about a pain in the back? Best Pract Res Clin Rheumatol. 2013;27(5):613–23.

17. Rundell SD, Gold LS, Hansen RN, Bresnahan BW. Impact of co-morbidities on resource use and adherence to guidelines among commercially insured adults with new visits for back pain. J Eval Clin Pract [Internet]. 2017 May; Available from: http://doi.wiley.com/10.1111/jep.12763

18. Hestbaek L, Leboeuf-Yde C, Manniche C. Is low back pain part of a general health pattern or is it a separate and distinctive entity? A critical literature review of comorbidity with low back pain. J Manipulative Physiol Ther. 2003 May;26(4):243–52.

19. Harris CA, D’Eon JL. Psychometric properties of the Beck Depression Inventory--second edition (BDI-II) in individuals with chronic pain. Pain. 2008;137(3):609–22.

20. Plummer F, Manea L, Trepel D, McMillan D. Screening for anxiety disorders with the GAD-7 and GAD-2: a systematic review and diagnostic metaanalysis. Gen Hosp Psychiatry. 2016 Mar 1;39:24–31.

21. Germans S, Van Heck GL, Hodiamont PPG. Results of the search for personality disorder screening tools: clinical implications. J Clin Psychiatry. 2012 Feb;73(2):165–73.

22. Andersen TE, Hansen M, Ravn SL, Seehuus R, Nielsen M, Vaegter HB. Validation of the PTSD-8 Scale in Chronic Pain Patients. Pain Med Malden Mass. 2018 Jul 1;19(7):1365–72.

23. Alsaadi SM, McAuley JH, Hush JM, Bartlett DJ, Henschke N, Grunstein RR, et al. Detecting insomnia in patients with low back pain: accuracy of four self-report sleep measures. BMC Musculoskelet Disord. 2013 Jun 27;14:196.

24. Takasaki H, Treleaven J. Construct Validity and Test-Retest Reliability of the Fatigue Severity Scale in People With Chronic Neck Pain. Arch Phys Med Rehabil. 2013 Jul 1;94(7):1328–34.

25. Beneciuk JM, Bishop MD, Fritz JM, Robinson ME, Asal NR, Nisenzon AN, et al. The STarT back screening tool and individual psychological measures: evaluation of prognostic capabilities for low back pain clinical outcomes in outpatient physical therapy settings. Phys Ther. 2013 Mar;93(3):321–33.

26. Hill JC, Dunn KM, Lewis M, Mullis R, Main CJ, Foster NE, et al. A primary care back pain screening tool: identifying patient subgroups for initial treatment. Arthritis Rheum. 2008;59(5):632–41.

27. Osman A, Barrios FX, Gutierrez PM, Kopper BA, Merrifield T, Grittmann L. The Pain Catastrophizing Scale: Further Psychometric Evaluation with Adult Samples. J Behav Med. 2000 Aug 1;23(4):351–65.

28. Coons MJ, Hadjistavropoulos HD, Asmundson GJG. Factor structure and psychometric properties of the Pain Anxiety Symptoms Scale-20 in a community physiotherapy clinic sample. Eur J Pain. 2004 Dec 1;8(6):511–6.

29. Roelofs J, van Breukelen G, Sluiter J, Frings-Dresen MHW, Goossens M, Thibault P, et al. Norming of the Tampa Scale for Kinesiophobia across pain diagnoses and various countries. PAIN. 2011 May 1;152(5):1090–5.

30. Neblett R, Mayer TG, Hartzell MM, Williams MJ, Gatchel RJ. The Fear-avoidance Components Scale (FACS): Development and Psychometric Evaluation of a New Measure of Pain-related Fear Avoidance. Pain Pract Off J World Inst Pain. 2016 Apr;16(4):435–50.

31. Miles CL, Pincus T, Carnes D, Taylor SJC, Underwood M. Measuring pain self-efficacy. Clin J Pain. 2011 Jun;27(5):461–70.

32. Hallegraeff JM, van der Schans CP, Krijnen WP, de Greef MH. Measurement of acute nonspecific low back pain perception in primary care physical therapy: reliability and validity of the brief illness perception questionnaire. BMC Musculoskelet Disord. 2013 Feb 1;14:53.

33. Jensen MP, Keefe FJ, Lefebvre JC, Romano JM, Turner JA. One- and two-item measures of pain beliefs and coping strategies. Pain. 2003 Aug 1;104(3):453–69.

34. Catley MJ, O’Connell NE, Moseley GL. How Good Is the Neurophysiology of Pain Questionnaire? A Rasch Analysis of Psychometric Properties. J Pain. 2013 Aug 1;14(8):818–27.

35. Swinkels-Meewisse EJCM, Swinkels RAHM, Verbeek ALM, Vlaeyen JWS, Oostendorp RAB. Psychometric properties of the Tampa Scale for kinesiophobia and the fear-avoidance beliefs questionnaire in acute low back pain. Man Ther. 2003 Feb;8(1):29–36.

36. Sullivan MJL, Adams H, Horan S, Maher D, Boland D, Gross R. The Role of Perceived Injustice in the Experience of Chronic Pain and Disability: Scale Development and Validation. J Occup Rehabil. 2008 Sep 1;18(3):249–61.

37. Holzapfel S, Riecke J, Rief W, Schneider J, Glombiewski JA. Development and Validation of the Behavioral Avoidance Test-Back Pain (BAT-Back) for Patients With Chronic Low Back Pain. Clin J Pain. 2016 Nov;32(11):940–7.

38. Hasenbring MI, Hallner D, Rusu AC. Fear-avoidance- and endurance-related responses to pain: development and validation of the Avoidance-Endurance Questionnaire (AEQ). Eur J Pain Lond Engl. 2009 Jul;13(6):620–8.

39. Fuhro FF, Fagundes FRC, Manzoni ACT, Costa LOP, Cabral CMN. Örebro Musculoskeletal Pain Screening Questionnaire Short-Form and STarT Back Screening Tool: Correlation and Agreement Analysis. Spine. 2016 Aug 1;41(15):E931-936.

40. Marhold C, Linton SJ, Melin L. Identification of Obstacles for Chronic Pain Patients to Return to Work: Evaluation of a Questionnaire. J Occup Rehabil. 2002 Jun 1;12(2):65–75.

41. Neblett R, Hartzell MM, Mayer TG, Cohen H, Gatchel RJ. Establishing Clinically Relevant Severity Levels for the Central Sensitization Inventory. Pain Pract. 2017 Feb 1;17(2):166–75.

42. Tanaka K, Nishigami T, Mibu A, Manfuku M, Yono S, Yukioka M, et al. Cutoff Value for Short Form of Central Sensitization Inventory. Pain Pract Off J World Inst Pain. 2020 Mar;20(3):269–76.

43. Georgopoulos V, Akin-Akinyosoye K, Zhang W, McWilliams DF, Hendrick P, Walsh DA. Quantitative sensory testing and predicting outcomes for musculoskeletal pain, disability, and negative affect: a systematic review and meta-analysis. Pain. 2019 Sep;160(9):1920–32.

**Supplementary Material 2: Initial version of the PDDM rating scale**

**Item 1: Nociceptive pain drivers**

**O** : □ The patient reports no symptom of pain.

**A** : □ Pain is influenced according to a mechanical pattern (e.g. reproduced during trunk movements, pain that increases during the day, stereotyped pain) and the patient responds to the criteria of a classification system among the following list: i) symptom modulation, ii) movement control, iii) functional optimization.

**B** : □ The patient DOES NOT respond to the criteria of a classification system.

**OR**

□ The patient seems to suffer from an active inflammatory process (e.g. Ankylosing Spondylitis flare) and/or the painful symptoms are not in relation with any mechanical pattern.

**OR**

□ The patient had a recent (less than three months) lumbar surgery or trauma (e.g. fracture) or presents disuse/deconditioning (e.g. global muscle deficits, sedentary patient).

**Item 2:** **Nervous system dysfunction drivers**

| **O** : □ No sign or symptom.  **A** : □ The patient shows painful symptoms as burning pain or electric shock which irradiate within a radicular pain pattern and/or paresthesia/tingling in a specific dermatome.  **OR**  □ □ The patient presents with a radiculopathy or myelopathy with at least 1 positive sign on neurological assessment:  - myotomes,  - dermatomes,  - loss of osteo-tendinous reflex and/or neurogenic claudication.  **B** : □ The physical examination revealed evidence of hypersensitivity by the presence of at least 1 element of the following list:  - Allodynia,  - Evidence of disproportionate pain intensity,  - Hyperalgesia,  - Widespread pain,  - Sympathetic nervous system dysfunctions (sweating, trophic or temperature changes in the skin),  - A score ≥40 on the Central Sensitization Inventory-25 items OR a score ≥20 on the Central Sensitization Inventory -9 items.  **OR**  □ The patient presents evidence of increased neural mechanosensitivity (i.e. SLUMP test, PKB test or SLR test).  **OR**  □ Pain disturbs patient’s sleep (important sleep disturbances directly related to the painful symptoms). |
| --- |

**Item 3: Comorbidity factors**

**O** : □ No comorbidity.

**A** : □ The patient reports ongoing painful comorbidity factors related to the musculoskeletal system (osteoarthritis, rheumatoid, tendinopathy, muscle or ligament tears).

**OR**

□ The patient reports pathology related to chronic pain (fibromyalgia, restless legs, migraine, complex regional pain syndrome, irritable bowel).

**B** : □ The patient reports sleep disorders not related to the actual pain (ex: insomnia, sleep apnea).

**OR**

□ The patient reports a diagnosis related to mental health disorder (depression, anxiety disorder, personality disorder, substance abuse or other DSM-5 diagnoses) and/or post-traumatic stress disorder.

**Item 4: Cognitive-emotional drivers**

| **O** : □ score ≤3 on the STart Back Screening Tool and the patient has no relevant maladaptive pain behaviors  **A :** □ Score >3 on the STart Back Screening Tool.  **AND**  □ The assessment (history and physical examination) highlights at least 2 "maladaptive" elements from the cognitive-emotional domain among:  - Pain-related anxiety,  - Pain-related fear OR fear of movement (kinesiophobia),  - Maladaptive beliefs about pain,  - Passive or harmful coping,  - Low level of understanding of one's health condition,  - Low perceived treatment efficacy,  - Low self-efficacy,  - Perception of injustice related to his situation,  - Negative anticipation of the evolution of his condition,  - High level of catastrophization,  - Strong perception of "being sick".  **B** : □ The patient exhibits maladaptive behaviors related to pain such as:  - Avoidance of certain tasks/movement,  - Facial expressions (e.g. grimacing, wincing during movement),  - A guarded posture,  - Moving as a block,  - Verbal/paraverbal pain expressions (e.g. grunts, sighs). |
| --- |

**Item 5: Contextual drivers**

**O** : □ No occupational or social factor identified.

**A :** □ score >49 on the Örebro

**OR**

□ The patient is on sick leave (total or partial) (or unable to participate in one of his/her occupations if he/she is retired or unemployed) and foresees obstacles for his/her return-to-work/occupation such as:

- Rigid internal return-to-work/occupation regulations,

- Perception of heavy work/occupation,

- High job/occupation stress,

- Low job/occupation satisfaction.

**B** : □ The patient CANNOT rely on the presence of a social support (family and friends) or professional (employer and colleagues) or caregivers facilitating his/her rehabilitation process (positive and empathetic attitude towards his/her condition)

**OR**

□ The patient is confronted with at least one element related to the social context among the following list:

- Negative experience during a previous episode of care,

- Restricted or non-existent access to the required care,

- Communication barriers (e.g., language, culture, difficulty telling his/her medical history).
