# Supplementary material for: Development and content validity of a rating scale for the pain and disability drivers management model
Source: Arch Physiother. 2022 May 16;12:14. doi: 10.1186/s40945-022-00137-2 (PMC9107946; doi:10.1186/s40945-022-00137-2)
Supplement: Supplementary file 2 — Additional file 2. [file 40945_2022_137_MOESM2_ESM.docx]

**Annex 1:** Description of the workshop.

The workshop consisted of a one-day (eight-hour) training, which focused on three topics:

(1) Knowledge of the relevance, content of the PDDM model and the definition of the different elements (3 hours).

(2) Ability to collect data for each domain and the different tools and procedures to use. We presented the rating scale and its utilization during this 3-hour segment.

(3) Analysis of the patient’s profile and establishment of a personalized treatment plan according to the patient’s profile (2 hours).
